# Pattern of Paediatric Neurological Disorders in Port Harcourt, Nigeria

**Published:** 2011-06

**Authors:** A. I. Frank-Briggs, E. A. D Alikor

**Affiliations:** 1 *Department of Paediatrics, University of Port Harcourt Teaching Hospital, Port Harcourt, Nigeria;*; 2 *Department of Paediatrics, University of Port Harcourt Teaching Hospital, Port Harcourt, Nigeria*

**Keywords:** neurological disorders, cerebral palsy, epilepsy, Nigeria

## Abstract

**Background:**

Paediatric Neurological disorders in developing countries are very challenging. This is due to its chronicity, late presentation and unavailability of modern diagnostic facilities in developing countries like Nigeria. Lack of these modern technology and manpower contribute significantly to increased morbidity and mortality. This study demonstrates the pattern of neurological disorders and the challenges in management in a developing country.

**Materials and Method:**

This was a retrospective hospital based analysis of neurological disorders seen in the Paediatric neurology unit of the University of Port Harcourt Teaching hospital, Nigeria from January 2004 to December 2009. Descriptive statistics was used to present the result.

**Result:**

A total of 35,473 patients were seen in the Paediatric unit. Of these 2,379 had neurological disorders. This gave a prevalence of 6.7% of Paediatric neurological disorders. There were 1,431 males and 948 females (male: female ratio of 1.51:1.0). The age ranged from 3 months to 15 years. The age group 1->5 years accounted for the most affected age group constituting 87.7%. The most frequent Paediatric neurological disorders were epilepsy (24.6%), cerebral palsy (15.4%), and central nervous system infections (9.5%).

**Conclusions/Recommendation:**

Wide spectrum of neurological disorders occur in our environment. The high incidence of epilepsy and cerebral palsy suggests that effort should be geared towards educating the populace about early diagnosis and prompt management.

## INTRODUCTION

Neurological disorders in children are common occurrence in clinical practice. The disorder account for more than 20% of the world’s disease burden with a greater majority of people affected living in Africa (1, 2). When affected by neurological disorders, a person’s memory, motor and cognitive abilities, concentration, speech, and physique can be drastically altered. Many of these disorders are chronic, frustrating to caregivers and parents and require adequate understanding to cope with management. The clinical manifestation of neurological disorders may progress and get worst over time. Not only do the people who live with these disorders suffer, their loved ones also experience great pain. Additionally, some affected children are unable to take care of themselves, such as feeding, clothing, and engaging in other basic everyday activities. Empirical observation suggests that the general attitude of parents of children with chronic illnesses such as neurological disorders in developing countries is to shop from one doctor to another in search of a cure or solution, this gives room for frustration as they usually end up being treated by non specialists. The treatment period may be so long taking months to years, making room for a high rate of default from follow-up (3).

In more developed countries, advances in diagnostic techniques have aided the characterization and definition of diseases. Also, application of recent therapeutic measures has resulted in significantly improved outcome (4). This is not the case in many developing countries. There is a rising trend in the incidence of neurological disorders in many developing countries contributed by lifestyle changes and HIV/AIDS pandemic. The challenge of managing these cases in a resource limited setting such as ours is burdensome. This retrospective study aims to evaluate the pattern of neurological disorders seen in Port Harcourt, Nigeria.

## MATERIALS AND METHOD

This was a retrospective survey of children who were seen in the Paediatric Neurology unit of the University of Port-Harcourt Teaching Hospital, Nigeria from January 2004 to December 2009. The patients studied were both out-patients and in-patients. Port Harcourt is the centre of the famous Nigerian oil industry with a population of 5, 410,115 and an area size of 21,850 Km. The teaching hospital is the only tertiary hospital located in the metropolis of Port Harcourt, the capital of Rivers State, one of the Niger Delta states in Nigeria. It is a 500-bed hospital and serves as a referral centre for hospitals within the state and neighboring states.

Data on age, gender, clinical history and examination findings, diagnosis and treatment outcome were collected from their case records. Children whose clinical conditions necessitated multidisciplinary evaluation were referred to the appropriate specialists in order to establish a definitive diagnosis and institute appropriate management. These included Ophthalmologists, Audiologists, Physiotherapists and Clinical Psychologists. Their case notes were coded in order to ensure that duplication did not occur. Common laboratory investigations available in our center and done included complete blood count, routine chemistries, blood, urine and stool cultures, and cerebrospinal fluid (CSF analysis). Ancillary investigations available included skull, spine and chest radiographs, computed tomography scan and magnetic resonance imaging studies. These tests were carried out for individual patients as needed. Electroencephalography (EEG) was also done for patients with seizure disorders. Data was analyzed using SPSS version 17 software and presented in frequency distribution tables and percentages.

## RESULTS

### General characteristics

During the study period, a total of 35,473 patients were seen in the Paediatric unit. Of these 2,379 had neurological disorders giving a prevalence of 6.7%. There were 1,431 males and 948 females (male: female ratio of 1.5:1). The age ranged from 3 months to 15 years. The age group 1->5 years accounted for the most affected age group constituting 87.7%. Those who were 10 years and above were least affected, accounting for 12.2%. The others are shown on Table 1.

**Table 1:** Age and gender distribution of the patients

Age range	Sex				Total
	Male	Percentage	Female	Percentage	

>1 year	151	10.6	124	13.1	275
1- >5 years	798	55.8	457	48.2	1255
5- >10 years	386	26.9	289	30.5	675
10 years and above	96	6.7	78	8.2	174
Total	1,431 (100)		948 (100)		

### Neurological diseases

Table 2 shows the different types of neurological diseases and their frequency. Seizure disorders (epilepsy) were the commonest neurological disorder accounting for about 25% of case. This was followed by cerebral palsy (15.4%). The different types of CP recorded included spastic quadriplegia 128 (5.4%), choreoathetoid type 104 (4.4%) from a combination of bilirubin encephalopathy and severe hypoxic ischemic encephalopathy; spastic hemiplegia 48 (2.0%), diplegic CP 21 (0.9%) and mixed type 66 (2.7%). There were 180 cases of microcephaly. The various causes of microcephaly were as follows: one hundred and five of them had hypoxic ischemic encephalopathy, two children had fetal alcohol syndrome, one had congenital rubella, 8 (0.3%) of them had craniosynostosis (Crouzon syndrome in 2 cases), the aetiology of the rest was unknown. Common features of those children with hydrocephalus included enlarged head size with craniofacial disproportion with sun set eyes, widely opened and bulging anterior fontanel, markedly dilated scalp veins, brisk tendon reflexes and spasticity of the limbs. Four (0.2%) of them had associated myelomeningocele (type II Chiari malformation). Another two had hydrancephaly with absence of the cerebral hemispheres. Majority of the cases of hydrocephalus were sequel to poorly or not treated meningitis. Out of the lot, only 43 (1.8%) of them had ventriculoperitoneal shunt inserted. Eleven (0.5%) opted for referral to other centers, 8 (0.3%) died; others were lost to follow up. The major complications seen were shunt infections, seizure disorder, and delayed motor development.

**Table 2:** Illustrates the different neurological disorders of the patients

	Number	Percentage

Epilepsy	584	24.6
Cerebral palsy	367	15.4
CNS infections	220	9.3
Microcephaly	180	7.6
Mental retardation	171	7.2
Learning disability	166	6.9
Hearing impairments	141	5.9
Speech disorders	120	5.0
Hydrocephalus	110	4.6
Neural tube defects	78	3.3
Brain tumors	56	2.4
Visual impairment	45	19
Behaviour disorders	42	1.8
Neuromuscular diseases	30	1.3
Neurocutaneous disorders	23	0.9
Chromosomal anomalies	16	0.7
Paralytic poliomyelitis	2	0.2
Guillian Barre syndrome	2	0.2
Others	26	1.1

Neural tube closure defect accounted for 78 (3.3%) of cases. The presentations included cranial encephalocele (nasofrontal type) in 2 (0.1%) cases, spinal bifida occulta 7 (0.3%) which manifested as tufts of hair in the sacral region in 3 of the children, sacral dimple in another 3 and one child with a 3 cm × 3 cm sized lumbosacral lipoma; and myelomeningocele in 69 (2.9%) of them. The myelomeningocele lesions were located in the lumbosacral region 54 (2.3%), mid lumbar 11 (0.5%), low sacral 3 (0.1%) and one had it in the cervical area. Out of the 69 children, only 23 of these children had surgical intervention within the first week of life. Twelve survived with quadriparesis as complication, they are still being followed up, 5 of them had post surgical hydrocephalus and 6 died within the first week post surgery. Seventeen preferred unorthodox therapy, 13 absconded from admission and the others refused surgical intervention.

The central nervous (CNS) infection included bacterial meningitis 156 (6.6%), tuberculous meningitis 17 (0.7%), and viral encephalitis (1.9%).

Brain tumor accounted for 56 (2.4%) of neurological disorders. Histological diagnosis of 6 (0.3%) of the cases revealed medulloblastoma in three and cerebellar astrocytoma in two children. Provisional diagnosis made for the others included craniopharyngiomas, colloid cyst and gliomas. Of the 23 cases that had neurocutaneous syndromes, 17 (0.7%) were diagnosed with neurofibromatosis, four (0.2%) had Tuberous sclerosis and two (0.1%) of them were diagnosed as having Sturge-Weber disease.

The neurobehavioral disorders included attention deficit hyperactivity disorder (ADHD) in 19 (0.8%), autism in 8 (0.4%), conduct disorder 6 (0.3%), post traumatic syndrome 5 (0.2%) and anxiety disorders in 4 (0.17%) cases. Those with speech disorders presented with selective mutism, stuttering, and inability to talk in the majority of cases. The causes were not identified.

Amongst the miscellaneous group, the neurological disorders were migraine (8), breath holding spells (5), Achondroplasia (3), carbon monoxide encephalopathy (3), post traumatic encephalopathy [head injury with neurological sequelae(3)], HIV encephalopathy (2), neurodegenerative disorder [Krabbe’s disease (1)] and prosencephaly (1). There were two children with HIV encephalopathy. The children presented with gross motor delay in one child (2 years old male); recurrent seizures and left sided hemiplegia in another 4 year old male.

## DISCUSSION

The finding from this study which showed that neurological diseases accounted for 6.7% of all Paediatric cases suggests that neurological disorders constitute a major cause of chronic morbidity in the Paediatric age group in Port Harcourt, Nigeria. Unfortunately, neurological disorders have not been given the desired attention in the developing world (6). This may be due to its chronicity and multidisciplinary management. Children between the ages of one to five years constituted more than two thirds of the cases seen in this study. This may be because most of the clinical manifestations of neurodevelopmental disorders become evident within this time frame when development is at its peak. Additionally, this group of children is very vulnerable to infections and their attendant high morbidities and mortalities (6).

The commonest neurological disorders in this study included epilepsy, cerebral palsy and CNS infections with its complications; they constituted 49.2%. This is similar to reports from other parts of the country (7, 8) and elsewhere in Africa (1). Also, Osuntokun in 1971 reported CNS infections, epilepsy, cerebral palsy, and poliomyelitis as the major paediatric neurological disorders seen at the UCH, Ibadan, Nigeria (9). These diseases still remain major causes of chronic morbidity in the Paediatric age group. This pattern is consistent with previous reports (8, 10). It is important to note that four decades after, the disease pattern has not changed. No new reports are available to the best of the authors research.

Epilepsy/seizure disorder was the commonest neurological disorder seen and managed in the neurology unit. This is similar to reports by other authors (11, 12). This high prevalence of epilepsy recorded may be due to increasing awareness that epileptic seizure is a medical condition which is treatable as against prior believe that it is caused by evil spirit manipulation and witchcraft attacks (11). It is possible that public enlightenment has contributed to its knowledge on aetiology and treatment modalities; thus, many more parents come to the hospital with their children for proper diagnoses and treatment.

Cerebral palsy (CP) was the second commonest neurological conditions from this study. This high number buttresses the fact that antenatal and perinatal medical care in our environment are still not at their best. Delivery in developing countries like Nigeria is taken by untrained traditional birth attendance, self help at home or in churches in the rural areas and even in some of the urban cities. These practices predispose to increased preventable risk/aetiological factors which contribute to high mortality rate. The risk factors include severe birth asphyxia, severe neonatal jaundice, low birth weight and intracranial infections (13, 14). CP imposes considerable economic, physical and psychological stress on the child and the affected family (15, 16).

Jaundice is frequently managed by exposing the affected child to early morning sunlight and use of glucose drink which has been shown to have little or no beneficial effect (17). These unorthodox modalities of care contribute immensely to cerebral palsy and associated neurological sequelae.

Central nervous system infections also contributed highly to the neurological problems in this study. This finding was similar to findings in earlier works done (18). Lack of medical personnel and preference for unorthodox treatments like spiritual healing are still conditions that militate against the drastic reduction of CNS infections in our society. The administration of routine childhood immunisations has markedly diminished the incidence of these infections in the developed world. This is not so in our setting as these vaccines, namely Haemophilus influenzae Type B vaccine and conjugate pneumococcal vaccine are currently not part of our national immunization programme due to the exorbitant cost (19).

Although preventable causes are responsible for the majority of the Paediatric neurological disorders in this part of the world, genetic disorders also played a role which cannot be overlooked similar to another report (20). Congenital CNS anomalies and other syndromes accounted for a small proportion of childhood neurological disorders in this study. This may be due to unavailability of hi-tech diagnostic equipments which could assist in making early and definitive diagnosis in our setting.

Seizure disorder and cerebral palsy contributed significantly to the number of neurological disorders seen in our center just as is reported in other parts of the world (11, 21, 22). Many of the affected children require specialized care and rehabilitative services.

### Limitation of the study/challenges

Difficulty in making definitive diagnosis in some brain tumors was a challenge. Very few patients with brain tumors had definitive diagnosis because of financial constraints; patients have to pay out of pocket for all investigations. The hospital has only one Paediatric neurosurgeon and the patients load is enormous. Also, treatment of these neurological disorders takes a long time and some of the patients were lost to follow up. Home visit for some of these cases could help.

## CONCLUSION

Neurodevelopmental disorders in Port Harcourt, Nigeria, contribute significantly to chronic morbidity amongst the children seen in the Paediatric unit. Continuing education of health workers and traditional birth attendants about the prevention of possible aetiological factors like asphyxia, infections, and jaundice need to be emphasized. Training and re-training of manpower to identify and institute prompt management of the various causes will significantly reduce the prevalence and aftermath of neurodevelopmental disability. Also, provision of biochemical diagnostic equipments to assist in the definitive diagnosis of genetic and chromosomal anomalies amongst others will go a long way. Advocacy for children and political will are needed to achieve these goals.
